# Cases of Recovery from Poisoning with Chloride of Zinc, and the Suggestions of an Antidote for This Poison

**Published:** 1849-02

**Authors:** T. Stratton

**Affiliations:** Edinburgh. Surgeon, Royal Navy, Particular Service; Member of the Natural History Society


### CHEMISTRY.

*Cases of Recovery from Poisoning with Chloride of Zinc, and the suggestions of an antidote for this Poison.*—By T. Stratton, M. D., Edinburgh. Surgeon, Royal Navy, Particular Service; Member of the Natural History Society.

When chloride of zinc is exhibited internally, its medicinal dose is from half a grain to two grains, two or three times a day. The following cases of swallowing in mistake, a quantity of a solution of chloride of zinc, lately occurred in Montreal.

Case 1. In a house in Craig street, in which I had been residing, there was a quart bottle, suitably labelled, containing a weak solution of chloride of zinc. E. R., a servant girl, aged 17, supposing that the bottle contained whiskey, put its mouth to her lips and (Nov. 4, 1847) drank about a wine-glass full. She instantly knew she had made a mistake; she experienced pain and nausea, and had a quantity of milk given her; she vomited very freely. She felt indisposition and want of appetite for about three weeks after. She was not seen by any medical man, as shame prevented her from speaking of the occurrence till a month after, when I saw her. On the supposition that she drank two ounces of the solution, I have reason to think that she took twelve grains of chloride of zinc.

Case 2. In May 4, 1848, J. C., aged 54, a porter, a stout healthy man, at noon took up a quart bottle, properly labelled, containing a dense solution of chloride of zinc, and supposing that it contained whiskey, he put it to his mouth and drank (as he afterwards told me, he supposed) about a wine-glass full. A large wine-glass contains two ounces and five drachms, and if we consider that he swallowed about two ounces of the solution, I have reason to think that he took four hundred grains of the chloride of zinc; but, from the nature of the liquid, it perhaps is unlikely that he took more than an ounce of the solution, or two hundred grains of chloride of zinc; from the size of the mouth of the bottle, it is not likely that he took less than this.

He instantly felt burning pain in the gullet, burning and griping pain in the stomach, great nausea, and a sense of coldness. In about two minutes he left the house, and vomited freely in the street for about fifty yards, till he came to a friend's house, where he lay down and continued to vomit, or endeavoured to do so. I was requested to see him, and I arrived about twenty minutes after; there was severe twisting and burning pain in the stomach; nausea and vomiting; cold-sweating; pulse 45, small, weak; his legs drawn up; anxiety and alarm. I instantly made a strong solution of home-made brown soap, and gave him a quantity of it. He vomited every two or three minutes, and in the intervals drank of the soap-suds, of which he had altogether three or four pints. He also had warm water. The matter vomited was quite free from odour, as I showed to Dr. Winder and Dr. Mount, who were present. He now felt much easier; there was not much stomach-pain, except on pressure; pulse 50; less coldness. I sent him home in a cab, in which he vomited at intervals all the way. I ordered twelve leeches to the epigastrium, and an ounce of olive-oil every hour.

Five P. M.—Has vomited several times after the olive-oil; pulse 60, natural fulness, soft, weak: tongue moist; no particular thirst. They could not procure leeches. A sinapism to the epigastrium. To take an ounce and a half of castor-oil now, and half an ounce of olive-oil every second hour.

May 5.—Slept a little; stomach is easier, still some heat and pain on pressure; he applied a second sinapism, which gave great relief; has vomited several times, soon after taking the olive-oil; tongue dry; thirst; one fœtid stool; pulse 72, soft. Repeated the castor-oil; continue the olive oil every four hours; linseed tea and water for drink; no food; a blister five inches square to the epigastrium. In the afternoon, he vomited four pieces, about three-quarters of an inch square, of a thin substance; they were not kept, but from the description they probably were eroded shreds of the mucous coat of the stomach.

May 6.—Blister rose well; no pain internally; tongue red on tip, brown on edges; pulse 80, small, soft, weak; thirst; two fœtid stools. No vomiting; discontinue the olive-oil; cold water only for drink; to take an ounce of castor-oil in the morning.

May 7.—got up: no pain on pressure over the abdomen; no vomiting; three fœtid stools; some appetite; pulse 60; tongue moist, white; weakness.

May 10.—Appetite pretty good; no uneasiness in the stomach. 12th: appetite improving. May 15: appetite, digestion, and strength, are the same as usual. May 30: he continues in perfect health.

On the first day, the patient was seen also by Drs. Winder, Mahony, Hall, and Mount; and several times after by Dr. Winder.

Remarks.—As the solution of the chloride of zinc was not made by myself, but supplied to me, I am not quite certain of its strength; I have good reason, however, to think that its strength is what I have stated above. The first patient took some of a diluted solution, and it is worthy of notice that she suffered from anorexia, &c., for three weeks after; while the second patient, who took a much larger dose, recovered his usual appetite in much less time; probably from his having administered to him the proper antidote, while the other did not apply at all for advice.

As chloride of zinc has a great deodorizing power, I took the opportunity of observing, in the second case, that the matter vomited had no odour, which probably arose from chloride of zinc. I was careful to observe if the stools were fœtid, and their being so was perhaps some proof that none of the chloride had passed lower than the stomach.

Antidotes.—Some time ago, on washing my hands with soap, after having had them in chloride of zinc solution, I observed that decomposition took place; and I thought, in the event of any one swallowing in mistake, or otherwise, an overdose of the chloride, that either soap, or carbonate of potash, or corbonate of soda, would be the proper antidote.

To a clear solution of chloride of zinc, I added a clear solution of carbonate of soda; carbonate of zinc was precipitated, and chloride of sodium, or common salt, remained in solution.

To a clear solution of chloride of zinc, I added a clear solution of carbonate of potash; carbonate of zinc was precipitated, and muriate of potash remained in solution.

To a clear solution of chloride of zinc, I added a solution of soap; the oil, or fat, in the soap, became free, and floated in the mixture in round and oval pieces; carbonate of zinc was precipitated, and muriate of potash remained in solution.

With regard to the requisite *quantity* of the antidote:—as soon as an overdose of chloride of zinc enters the stomach, one of its first effects, fortunately, is an emetic one; but perhaps cases will occur where, from an overloaded state of the stomach, or some other cause, vomiting will not have occurred by the time the physician reaches the patient; in such cases, for a drachm of chloride of zinc, the proportional antidotal dose is either a drachm of the carbonate of soda, or a drachm and a half of carbonate of potash, or as much soap as contains the above quantities of soda or potash. (In soap there is generally from six to ten per cent. of either soda or potash.) In nearly all cases, it will probably be found, that vomiting will occur immediately after taking the poison, so that much less than the above quantities of antidote will suffice. It is exceedingly convenient to possess an antidote in soap, which is always to be had in houses without delay. Even when soda or potash is at hand, as well as soap, the last seems preferable, as its oily part is useful either as an emetic, or to soothe the irritated or abraded mucous membrane. Castor-oil may be prescribed to carry off any of the chloride which may have passed the stomach. Olive-oil for a day or two, is soothing to the mucous lining of the œsophagus and stomach, and sinapisms or a blister to the epigastrium appear to be all that is required.

Chloride of zinc, in medicinal doses, is useful in chorea, neuralgia, epilepsy, &c.; in surgical practice it is used as a caustic and escharotic, and applied externally in a weak solution, it possesses stimulant, alterative, and deodorizing powers over certain ulcers, where it has the great advantage over arsenical, mercurial, and lead preparations, of never giving rise to constitutional disorder from absorption. A peculiar solution of it (Sir William Burnett's Disinfecting Fluid) is largely used to preserve timber, canvass, and cordage, from decay, and to preserve anatomical preparations, and for its deodorizing and disinfecting properties, and for various other hygienic purposes; and this solution, used in the manner directed, is perfectly innocuous.

I have looked into seven or eight of the latest works on Materia Medica and Toxicology, and have not found mention made of any antidote for chloride of zinc; in one of these works, there is, in parallel columns, a list of poisons and their antidotes; and that for chloride of zinc is left blank; so that, as far as I know, I am the first who has pointed out, and who has used the proper antidote for this poison.—*Brit. Am. Jour. of Med. and Phys. Sci.*

